# Cholestasis in patients with Cockayne syndrome and suggested modified criteria for clinical diagnosis

**DOI:** 10.1186/1750-1172-6-13

**Published:** 2011-04-08

**Authors:** Tawhida Y Abdel Ghaffar, Ezzat S Elsobky, Solaf M Elsayed

**Affiliations:** 1 Yassin Abdelghaffar Charity Center for Liver Disease and Research, Cairo, Egypt; 2 Children's Hospital, Ain Shams University, Cairo, Egypt; 3 Medical Genetics Center, Cairo, Egypt

## Abstract

**Background:**

Cockayne syndrome is a rare autosomal recessive neurodegenerative disease characterized by low-to-normal birth weight; growth failure; brain dysmyelination with calcium deposits, cutaneous photosensitivity; pigmentary retinopathy, cataract, and sensorineural hearing loss. To the best of our knowledge, cholestatic liver disease was not previously reported in these patients.

**Aim:**

To highlight the presence of cholestasis and liver dysfunction in this group of patients and to suggest modified criteria for clinical diagnosis.

**Methods:**

The study included nine patients with Cockayne from four different families (five males and four females) in which Cockayne was suspected clinically. In all patients chromosomal breakage studies revealed mild (45%) to moderate (60%) increase in frequency of chromatid and chromosome gaps and breaks versus 25% in normal controls. Diagnosis was confirmed by DNA repair assay.

**Results:**

During routine follow up of these patients, seven of them had evident liver affection ranging from mild elevation in liver enzymes to cholestatic liver disease and liver cell failure. The attacks were recurrent in two patients and were sometimes preceded by infection. The attack may lead to deterioration of neurological and/or liver condition. It may end in liver cell failure that either recovers completely or may lead to death.

**Conclusions:**

liver disease could be considered common in Egyptian patients with Cockayne with the cholestatic form being the most evident. The syndrome should be included in the list of causes of cholestatic liver disease. Chromosomal breakage study and positive family history should be included as major criteria for clinical diagnosis of Cockayne especially in a population like ours where consanguineous marriage is very high and molecular testing and UV sensitivity tests are considered unaffordable.

## Background

Cockayne syndrome (CS) was first reported in 1936 in two siblings who were normal at birth but showed progressive mental retardation and had characteristic senile facies with closely spaced sunken eyes. The disease is characterized clinically by cachectic dwarfism, cutaneous photosensitivity, loss of adipose tissue, mental retardation, skeletal and neurological abnormalities, pigmentary degeneration of the retina and hepatomegaly [1]. Before the molecular genetics of CS was understood, it was thought that CS has a single, discrete phenotype: classic CS. Now it is recognized that this syndrome spans a spectrum that includes: CS type I (the classic form of CS), type II, a more severe form with symptoms present at birth (previously called cerebro-oculo-facial syndrome (COFS) and Pena-Shokeir type II syndrome); CS type III, a milder form; and xeroderma pigmentosum- Cockayne syndrome (XP-CS) [2]. To the best of our knowledge, cholestatic liver disease was not previously reported in these patients.

Definitive diagnosis of CS can be done by DNA repair assay [3] or molecular testing [4], both are expensive and done in few specialized laboratory worldwide.

The aim of this report is to highlight the presence of cholestasis in this group of patients and to suggest modified criteria for clinical diagnosis.

## Methods

### 1- Patients and families

#### Family (1)

**Figure 1:**
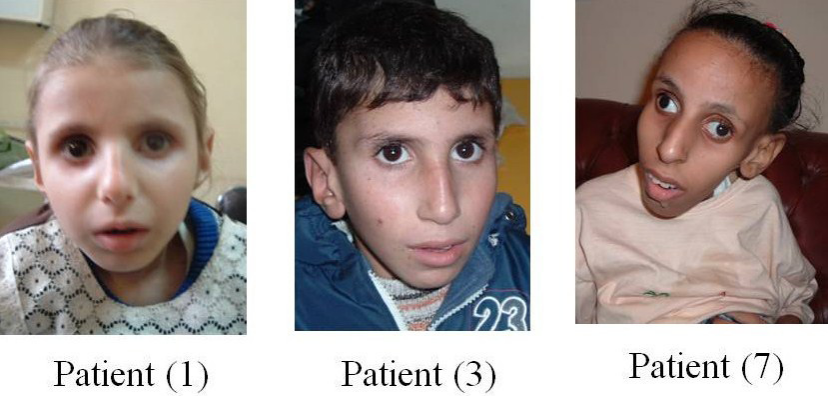
**Anteropesterior view of three patients in the studied group**.

**Figure 2:**
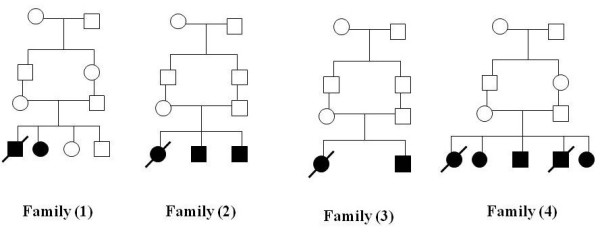
**Family pedigrees of the studied group**.

*Patient (1) *is a 3 years old female, the second in order of birth of a first cousin marriage (Figures 1 and 2). She was referred to the genetic clinic at the age of one year with a provisional diagnosis of congenital Rubella syndrome. At presentation she had jaundice of one week duration, her length was 71.5 cm (less than - 4 SD), weight was 7 Kg (less than - 4 SD), and skull circumference was 39 cm (microcephalic). She had global developmental delay (that progressed in the next years), enophthalmia with characteristic physical appearance and cutaneous photosensitivity. Abdominal examination revealed an enlarged liver (8 cm below the costal margin) firm in consistency, with rounded border and smooth surface. She had moderate ascites with bilateral shifting dullness and bilateral lower limb oedema.

ALT was 94 IU/L (normal: 37), AST: 143 IU/L (normal: 40), total bilirubin: 5.9 mg/dl, direct bilirubin: 5 mg/dl, albumin: 1.5 g/dl. Prothrombin time (PT) was prolonged with INR: 1.7. Hepatitis A virus IgM, HBsAg, HBcAb IgM, hepatitis C virus antibodies were negative; CMV, EBV and rubella (IgG and IgM) were also negative. Plasma aminoacids revealed a mild elevation of methionine: 1.3 mg/dl (normal < 0.8) and an elevation of methionine/phenylalanine ratio: 1.41 (normal < 1.0). Acylcarnitine profile was normal. Total galactose (galactose and galactose 1- phosphate combined): 1.7 mg/dl (normal <15). Galactose 1- phosphate uridyle transferase was: 71.2 uM (normal >60). DNA testing for galactosemia mutation revealed no copies of the Q188r, S135L, K285N, or L195P (classical galactosemia) or N314D (Duarte galactosemia).

Slit lamp examination revealed clear corneas and lens. Fundus examination showed pigments around the disc (bilateral retinal dystrophy). CT brain revealed central and cortical involutional brain changes. MRI brain revealed mild prominence of the brain sulci, fissures and basal cisterns with mildly dilated ventricular system more appreciated at the cerebellar hemispheres with mildly prominent cerebellar fissures. This is in addition to evidence of dysmyelinating process in the form of periventricular hyperintensity. Nerve conduction velocity and EMG showed normal studies.

The patient received ursodeoxycholic acid (UDCA) at a dose of 20 mg/kg/day, vitamin E (100 mg/day), aldactone (2 mg/kg/day), vitamin K (10 mg/day) and albumin transfusion. She gradually improved over one month and was discharged from the hospital on aldactone (12.5 mg/12 hrs) and vitamin K (10 mg once daily). Her investigations on discharge were: total bilirubin: 3.6 mg/dl, direct bilirubin: 2.1 mg/dl, albumin: 4 g/dl, AST: 54 IU/L, ALT: 100 IU/L, and normal PT. After 2 months, the amino acid profile was repeated and revealed normal results. At that time, all drugs were stopped and after 6 months the liver condition was completely normal with the mildly raised liver enzymes as the only abnormal finding.

*Patient (2) *was the older brother of patient (1). He died after a prolonged attack of jaundice at the age of 2 years before patient (1) presented to our clinic. According to the parents' history and the available reports, the patient had growth failure, similar characteristic features to his sister, delayed physical and mental milestones, microcephaly, cutaneous photosensitivity and cataract. Fundus examination showed pigmentary retinopathy.

#### Family (2)

*Patient (3) *is a 4 years old boy, the third in order of birth of first cousin marriage. He presented to the hepatology clinic with jaundice following an attack of vomiting and diarrhea. He had a similar attack at the age of two years that regressed spontaneously.

On examination the patient had progressive neurologic dysfunction, his height was 95 cm (-2 SD), weight was 13 kg (-3 SD) and skull circumference was 43 cm. He had characteristic physical appearance with enophthalmia, nystagmus, jaundice, and skin photosensitivity. His liver was 4 cm below the costal margin, firm in consistency with rounded border and smooth surface. The spleen was felt two cm below the costal margin. Neurological examination showed intention tremors, cerebellar ataxia, lost knee and preserved ankle deep reflexes. Chest and heart examination were clinically free.

Investigations at that time revealed ALT: 270 IU/L (N: 37), AST: 232 IU/L (N: 40) GGT: 183 (N: 50), total bilirubin: 3.35 mg/dl, direct bilirubin: 2.6 mg/dl, albumin: 3.2 g/dl, PT 11.7 sec. (INR: 1). Tests for HBV, HCV, and HAV revealed negative results. Ceruloplasmin level, protein electrophoresis and auto antibodies were also normal.

Abdominal ultrasonography revealed enlarged liver with regular border, homogenous echopatteren, patent portal and hepatic veins. Fundus examination showed no abnormality. CT brain was normal, while MRI brain showed deep white matter signal alteration at the forceps minor and major regions, middle cerebellar peduncles, the posterior segmental aspects of the pontine isthmus and basis pontis. Those areas exhibited bright signal on FLAIR and T2W1, a picture consistent with dysmyelinating disease.

The patient received ursodeoxycholic acid at a dose of 20 mg/kg/day and vitamin E (100 mg/day). Two months later, ALT decreased to 83 IU/L, AST decreased to 95 IU/L, total bilirubin was: 1.7 mg/dl, direct bilirubin: 0.7 mg/dl, albumin: 3.2 g/dl, and PT 11.3 seconds.

Six months later, the patient had another similar attack that was also precipitated by viral infection and was associated with increased tremors, ataxia and inability to walk. He received the same treatment with excellent response and complete recovery of liver functions, and improvement of ataxia, and tremors.

*Patient (4) *is a 13 years old boy (the older brother of patient (3)). His weight was 17.5 kg (less than - 4 SD), height was 115.5 cm (less than - 4 SD) and skull circumference was 50.5 cm. He had progressive neurologic dysfunction, characteristic physical appearance of the stooped standing appearance and senile facies, sunken eyes and skin photosensitivity. Neurological examination revealed typical cerebellar ataxia. He also had history of recurrent attacks of jaundice before his presentation to our clinic that were always preceded by infection. Bilirubin level, ALT, AST, INR and albumin were normal at time of presentation. Urine and plasma aminograms were also normal.

#### Family (3)

*Patient (5) *is a one month old boy, the second in order of birth of first cousin marriage. He presented to the genetics clinic with microcephaly (skull circumference: 31 cm), small anterior fontanel, deep seated eyes, polydactyly of both hands and left foot. CT brain was normal. His older sister *Patient (6)*: According to the parents' history and the medical reports, she had progressive neurological dysfunction, skin photosensitivity, microcephaly, enophthalmos, nystagmus, cataract, and spasticity of both lower limbs. Fundus examination revealed pigmentary retinopathy and CT brain showed calcification of basal ganglia. She developed jaundice after anaesthesia for cataract extraction at the age of 10 years and died soon after the operation as a result of hepatocellular failure.

#### Family (4)

*Patient (7) *is a 16 year old girl, the second in order of birth of first cousin marriage. She presented to the genetics clinic with severe growth retardation, progressive intellectual deterioration, deep seated eyes, bilateral cataract, and cerebellar ataxia. Her weight was 12.5 Kg (< 4SD), length was 120 cm (she could not stand), and skull circumference was 49.8 cm. Her abdominal examination revealed no organomegaly but her ALT was mildly elevated (46 IU/L, normal: 40). Nerve conduction velocity showed axonal neuropathic disease and audiometry showed moderate sensorineural hearing loss.

*Patient (8)*: is a 6 year old boy, the brother of patient (7). His height was 110 cm (< 4SD), his weight was 11.5 Kg (< 4SD) and skull circumference was 44 cm. He had the same characteristic physical features of his older sister with cataract and cerebellar ataxia. Abdominal examination revealed enlarged liver (palpable 4 cm below the costal margin). His chest and heart examination were clinically free.

Abdominal ultrasound showed mildly enlarged liver with normal spleen. ALT was 135 IU/L (normal = 40), AST was 126 IU/L (normal = 37) and GGT was 85 U/L (normal = 50). Nerve conduction velocity showed axonal neuropathic disease and audiometery showed moderate sensorineural hearing loss.

*Patient (9)*: is a 2.5 year old girl, the youngest sister of patient (7 and 8). Her height was 90 cm and her weight was 8.5 Kg and skull circumference was 42 cm. She had bilateral cataract that was extracted at the age of 1.5 years and cerebellar ataxia. Abdominal examination revealed mildly enlarged liver which was palpable 2 cm below the costal margin. Her chest and heart examination were clinically free. Abdominal ultrasound showed no abnormalities apart from the mildly enlarged liver. ALT was 101 IU/L (normal = 40), AST was 49 IU/L (normal = 37) and GGT was 27 U/L (normal = 50). Although bilirubin level in this family was normal, GGT was mildly increased in patient (8). Table 1 and 2 demonstrates patient clinical criteria and laboratory investigations at time of presentation.

**Table 1:** Patient criteria according to Nancy and Berry 1992 [2]

Clinical criteria		1	2	3	4	7	8	9
**Major criteria**	**Post natal growth failure**	+	+	+	+	+	+	+
	**Progressive neurological dysfunction**	+	+	+	+	+	+	+

**Minor criteria**	**Cutaneous photosensitivity**	+	+	+	+	-	-	-
	**Peripheral neuropathy**	-	ND	ND	ND	+	+	ND
	**Pigmentary retinopathy and/or cataract**	+	+	_	-	+	+	+
	**SNHL**	ND	?	ND	ND	+	+	ND
	**Dental caries**	+	?	+	+	+	+	+
	**Characteristic physical appearance**	+	+	+	+	+	+	+
	**Family history**	+	+	+	+	+	+	+

Patients 5 and 6 were excluded as the criteria do not apply to them (CS type II). ND: not done, SNHL: sensorineural hearing loss.

**Table 2:** Clinical and laboratory data of patients at presentation

***Patient No***.	1	2	3	4	5	6	7	8	9
***Age (years)***	3	2	4	13	0.8	10	16	6	2.5
***Sex***	F	M	M	M	M	F	F	M	F
***Jaundice***	+	+	+	-	-	+	-	-	-
***Hepatomegaly***	+	NAD	+	-	-	NAD	-	+	+
***splenomegaly***	-	NAD	+	-	-	NAD	-	-	-
***Ascites***	+	NAD	-	-	-	NAD	-	-	-
***Total bilirubin (mg/dl)***	5.9	NAD	3.35	N	N	NAD	N	N	N
***Direct (mg/dl)***	5	NAD	2.6	N	N	NAD	N	N	N
***ALT(XN)***	2.5	NAD	7.2	N	N	NAD	1.15	3.4	2.5
***AST (XN)***	3.5	NAD	5.8	N	N	NAD	0.7	1.7	1.3
***GGT (U/L)***	128	NAD	183	N	ND	NAD	30	85	50
***Albumin (gm/dl)***	1.5	NAD	3.2	N	N	NAD	N	N	N
***INR***	1.7	NAD	1	N	N	NAD	N	N	N

M: male, F: female, +: present, -: absent, NAD: No available data, N: normal, ND: Not done

### 2- Chromosomal breakage studies

**Figure 3:**
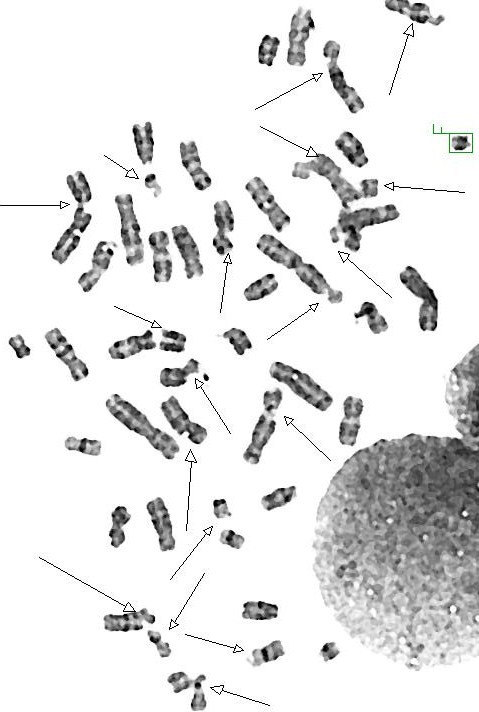
**Metaphase spread showing increase in frequency of chromatid and chromosome gaps and breaks in our patients with CS**.

All cases were subjected to both routine phytohemagglutinin stimulated peripheral blood cultures and chromosomal breakage study using mitomycin C and diepoxybutane independently according to previously described methods [5]. Karyotype was done from routine cultures to exclude constitutional rearrangements and to detect any spontaneous breakage. Harvesting and analysis of at least 20 metaphases from breakage induction cultures and scoring of numbers and types of induced chromosomal aberrations was also done. All metaphases from routine culture revealed normal karyotype excluding constitutional abnormalities. Chromosomal breakage studies revealed mild (45%) to moderate (60%) increase in frequency of chromatid and chromosome gaps and breaks versus 25% in normal control (Figure 3)

### 3- DNA recovery tests

DNA recovery tests were done to one patient in each family (patient number 1, 3, 5 and 7). In this test cultured fibroblasts were labeled with 14C thymidine to have a measure for the amount of cells in the dish (thymidine is a measure of DNA synthesis). Then the cells were irradiated with UV light and incubated further in fresh medium. Three UV doses were used (1, 3 and 6J/m2). After 17 hours, the incorporation of thymidine into the DNA was measured again. In normal cells DNA synthesis decreases strongly in the first few hours after UV but the rate begins to increase again after 3-6 hours and keeps recovering during the next 24 hours (depending on the dose). In CS cells DNA synthesis is strongly inhibited and does not recover at all.

**Figure 4:**
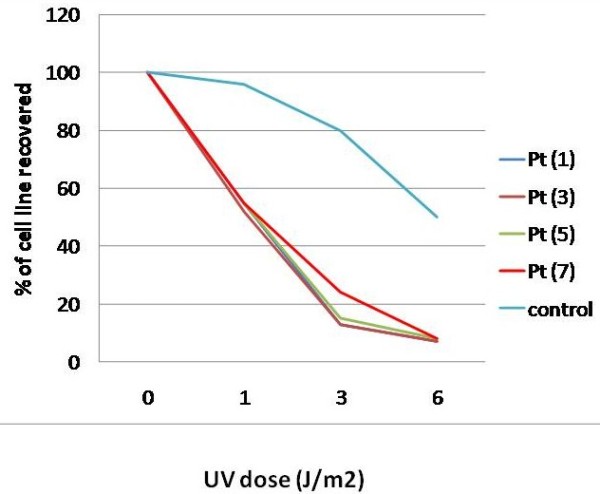
**Response of cells from patients to different doses of UV irradiation compared to controls**. There is strongly reduced recovery of DNA synthesis after UV-irradiation in our patients compared to the normal control.

In this study, cells from the four patients showed strongly reduced recovery of DNA synthesis after UV radiation, confirming the diagnosis of Cockayne syndrome (table 3, Figure 4)

**Table 3:** Strongly reduced recovery of DNA synthesis after 3J/m^2 ^of UV-irradiation in patients with CS compared to positive controls (known CS patients) and negative controls (normal individuals).

Patients	% of Recovery of DNA synthesis at 17 hrs after UV (3J/m^2^)		
	
	Our patients	Known CS patients	Normal controls
**Patient (1)**	13	14	80
**Patient (3)**	13	15	88
**Patient (5)**	15	22	74
**Patient (7)**	24	26	67

It can be noticed that normal cells can recover 67 to 88% of DNA compared to only 13-24% in patients with CS.

## Results and Discussion

Liver dysfunction was a prominent feature in our group of patients with CS (7/9 (77.8%)). Although they manifested different forms of liver affection, the most evident were hepatomegaly and recurrent attacks of cholestasis. To best of our knowledge, cholestatic liver disease was not described before although hepatomegaly and hepatic dysfunction as a complication of anesthesia were previously reported [6].

It was observed that our patients' attacks were preceded (or precipitated) by infection (patient 3 and 4), a stressful conditions (surgery) or by a possible hepatotoxic drug that was used during anesthesia (patient 6).

These attacks led to deterioration of the patient's liver condition and were associated with worsening of their neurological symptoms. In patient 3 and 4, the jaundice was followed by inability to walk or increased tremors and ataxia. It proceeded to liver cell failure that had recovered completely (patient 1), or ended in death as in (patients 2 and 6).

Although these attacks may have resolved spontaneously, vitamin E and UDCA were given to hasten their resolution and preserve the liver condition. Several mechanisms have been proposed to explain the potential beneficial effects of UDCA in the treatment of cholestatic liver disease. Because intracellular retention of hydrophobic bile acids is thought to injure liver cells, replacement of these compounds with a nontoxic hydrophilic bile acid such as UDCA should theoretically reduce injury. UDCA may be hepatoprotective by displacing toxic bile acids from both the bile acid pool and hepatocellular membrane [7].

The major function of vitamin E is its role as an antioxidant, protecting cell membrane polysaturated fatty acids and thiol-rich proteins from oxidant damage initiated by free-radical reactions [8]. As vitamin E deficiency may impair immune function and may worsen cholestatic liver injury [9], it was used in our patients with CS and cholestasis.

Considering the high consanguinity rate in our population, the presence of another recessive entity causing liver diseases in these patients would make sense especially that it was previously reported by our group [10]. The presence of cholestatic liver disease in 4 different families from 4 different regions in Egypt support that liver affection is a feature of CS and contradicts the above assumption.

Despite the various diagnostic tests, early diagnosis for CS is still problematic. This was obvious in all reported patients in this study (congenital Rubella syndrome in family (1), unidentified liver disease in family (2), and unknown genetic disease in families (3, 4)).

**Figure 5:**
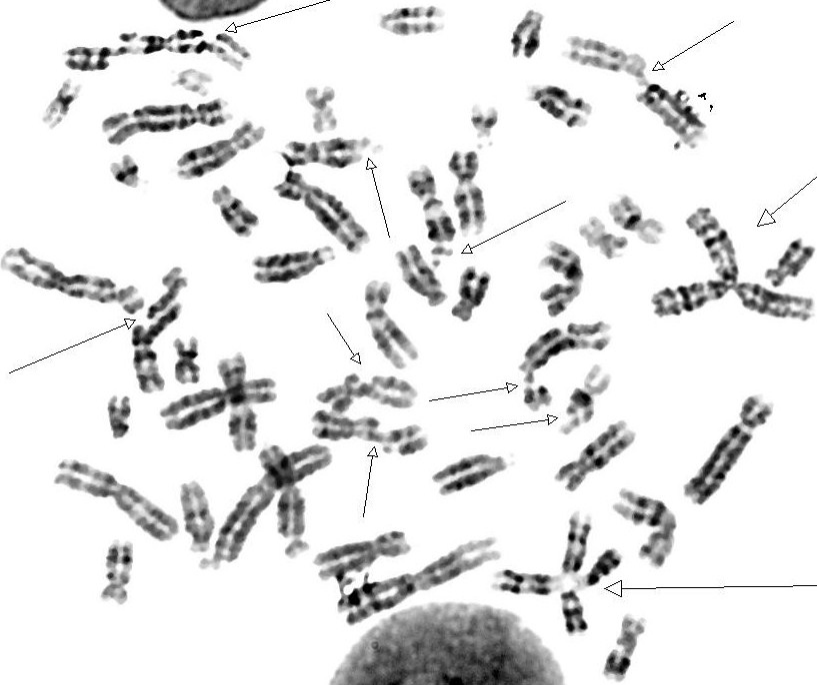
**Metaphase spread from a patientwith Fanconi anemia with a significant level of exchange and asymmetrical interchange noted**.

Clinical diagnostic criteria for CS type I were suggested by Nance and Berry, 1992 [2]. Seven of the patients were CS type I (they met these criteria) and two (patients 5 and 6) were CS type II. The diagnosis was highly suspected in the presence of an affected sib and by the presence of mild to moderate increase in chromosomal breakage using mitomycin C in all patients. This is in contrast to patients with Fanconi anemia where a very high breakage (90-100%) with a very significant level of exchange and asymmetrical interchange is usually noted (Figure 5).

Therefore, we suggest that the diagnostic criteria should be modified to include chromosomal breakage study (which is a simple inexpensive test) as a major criterion especially in very young patients in whom the diagnosis is extremely difficult and in a poor country like ours in which the DNA repair studies and molecular studies are extremely expensive. Also it should include positive family history especially in populations with high prevalence of consanguineous marriage like ours (35-50%) in which the affection of more than one member in the family is quite common [11]. This may be further supported by the variability in the clinical presentation even between members of the same family (table 1) which was previously reported by Pasquier et al, 2006 [12].

## Conclusions

Liver disease could be considered common in Egyptian patients with CS with the cholestatic form being the most evident. Stressful condition may precipitate these attacks and liver support with careful follow up is extremely essential during the attacks, together with avoidance of hepatotoxic drugs. Further studies on a larger number of patients are needed to investigate whether these observations are part of CS or a simple association in these extremely cachectic patients.

We suggest that the diagnostic criteria of CS should be modified to include chromosomal breakage study (which is a simple inexpensive test) and positive family history as major criteria especially in very young patients in which the diagnosis is extremely difficult and in a poor country like ours in which the DNA repair studies and molecular studies are extremely expensive.

## List of abbreviations

COFS: Cerebro-oculo-facial syndrome; CS: Cockayne syndrome; UDCA: ursodeoxycholic acid; UV: Ultraviolet; XP-CS: Xeroderma pigmentosum- Cockayne syndrome.

## Consent

Written informed consent was obtained from the parents of the patients for publication of this case report and accompanying images. A copy of the written consent is available for review by the Editor-in-Chief of this journal.

## Competing interests

The authors declare that they have no competing interests.

## Authors' contributions

TYA: diagnosed the liver disease, followed the patients, analyzed and interpreted the data, and participated in writing the manuscript.

ESE: carried out the chromosomal breakage study, diagnosed the genetic disease, analyzed and interpreted the data, and participated in writing the manuscript

SMS: diagnosed the genetic disease, followed the patients, analyzed and interpreted the data, and participated in writing the manuscript

All authors read and approved the final manuscript.
